# As to the Value to Physicians of the Summer Vacation

**Published:** 1884-10

**Authors:** 


### EDITORIAL.

As to the Value to Physicians of the Summer Vacation.

During the late absence from this city, of the senior editor of this journal, an anonymous article, for which he was not responsible, appeared in these columns. It was entitled, "The Samuel D. Gross Professorship of Pathological Anatomy," and contained some pointed reflections upon the position assumed on this same subject by the editor of the New York *Medical Record.*

Under date of September 20th, the *Medical Record* responds with certain editorial comments, apparently written in a retaliatory spirit, which we are charitable enough to hope were inspired when the editor of that esteemed cotemporary also was absent for the heated term from the city of New York.

There is such an obviously absurd side to this situation that it need scarcely be pointed out. We of course, offer our explanation to our editorial brother of the adjectives of which he complains, offensive to the spirit of courtesy which should characterize all discussions however remotely associated with scientific themes.

We are even charitable enough to accept in advance, with the utmost good nature, the expression of regret which our friend of *The Record* is certain to offer.

The lesson to be learned from this affair, is really deeper than that suggested by the writer on the staff of *The Record.* It seems to have occurred to him that the parties at either end of the line were composing mid-summer editorials with the atmospheric temperature at or near the nineties. He could think of nothing so valuable for his adversary as a packing in ice. Yet we all know that the temperatures thus artificially induced are apt to be followed by the exaggerated hyperæmias of a restored and active circulation.

We recommend rather to such fiery spirits, the rest and recreation of a vacation in the summer season. It is better at these times, for our Eastern friends to cultivate an acquaintance with the sea-shore; and for others the glades and lakes of our Western Wisconsin.

Returned from these peaceful associations, young gentlemen, ye may well ask yourselves whether it is either a pleasant or a profitable commerce to exchange disagreeable adjectives between Chicago and New York.

